# A high sensitivity assay is more accurate than a classical assay for the measurement of plasma CRP levels in endometriosis

**DOI:** 10.1186/1477-7827-9-113

**Published:** 2011-08-09

**Authors:** Alexandra Vodolazkaia, Xavier Bossuyt, Amelie Fassbender, Cleophas M Kyama, Christel Meuleman, Karen Peeraer, Carla Tomassetti, Thomas M D'Hooghe

**Affiliations:** 1 Leuven University Fertility Centre, Department of Obstetrics & Gynaecology, University Hospital Gasthuisberg, Leuven, Belgium; 2 Division of Reproductive Biology, Institute of Primate Research, Nairobi, Kenya; 3 Department of Laboratory Medicine, Leuven University Hospital, Leuven, Belgium; 4 Experimental Gynaecology Laboratory, Department of Obstetrics & Gynaecology, KU Leuven, University Hospital Gasthuisberg, Herestraat 49, B3000 Leuven, Belgium

## Abstract

**Background:**

Endometriosis is associated with chronic subclinical inflammation. C-reactive protein (CRP), a marker of inflammation, could serve as a biomarker of endometriosis. We tested the hypothesis that a high sensitivity CRP assay (hsCRP) is more accurate than a classical CRP assay in the detection of subclinical inflammation in plasma of women with endometriosis.

**Methods:**

CRP levels were measured by hsCRP and classical CRP assays in plasma of 204 women with endometriosis and 91 women without endometriosis. Both assays were compared with respect to their value for the diagnosis of endometriosis.

**Results:**

The number of plasma samples with detectable CRP was significantly higher (100%) using the hsCRP assay when compared to the classical CRP assay (42.7%) (p < 0.0001). Significantly increased CRP plasma levels were found in women with endometriosis when compared with controls when the hsCRP assay was used in samples obtained during the luteal phase (p = 0.008). The highest discriminative ability for the diagnosis of endometriosis was also obtained using the hsCRP assay during the luteal phase, especially for moderate -severe endometriosis. At a cut-off level of hsCRP > 0.71 mg/L, moderate-severe stages were diagnosed with 80.7% sensitivity and 63.9% specificity during the luteal phase. Using a similar cut-off value for CRP analyzed by the classical method, moderate-severe endometriosis was diagnosed with lower sensitivity (67.7%, p = 0.06) and comparable specificity (63.9%).

**Conclusions:**

The hsCRP assay was superior to the classical CRP assay for the detection of low CRP levels and for revealing subclinical inflammation in plasma of women with endometriosis.

## Background

Endometriosis is defined as the presence of endometrial-like tissue outside the uterine cavity, associated with a chronic, inflammatory reaction. Although the pathogenesis of endometriosis is still controversial, growing evidence indicates a significant role for immunological and inflammatory factors in the development of endometriosis [1] as demonstrated by increased concentrations of activated macrophages, cytokines, angiogenic factors, T cells and B cells [2-4].

Endometriosis can be considered as an inflammatory disease [5]. C-reactive protein (CRP) is an acute phase protein and a marker of inflammatory reaction, could serve as a potential non-invasive biomarker of endometriosis. Its production is stimulated by pro-inflammatory cytokines such as IL-6, IL-1 and TNF-alpha [6] which are up-regulated in women with endometriosis when compared to controls [1,7].

CRP is widely accepted as a biochemical marker of systemic inflammation [8] and routinely used as a marker of infection, inflammation or tissue damage in clinical practice [6]. Moreover, slightly elevated CRP levels may indicate a low level of chronic inflammatory reaction in patients at risk of developing metabolic syndrome, colon cancer and cardiovascular disease [9,10].

Published evidence suggested that endometriosis could be viewed as a local disease with systemic subclinical manifestation [7]. Data regarding the CRP level in peripheral blood of endometriosis patients are relatively scarce and controversial [11-14] probably related to differences in study design, patient selection and methodology used to detect CRP levels in peripheral blood. In our study, samples were collected at a well defined phase of the cycle and results were corrected for cycle phase, as recommended by the QUADAS (Quality Assessment of Diagnostic Accuracy Studies) guidelines [15,16].

Classical automated methods for CRP measurement typically have limited sensitivity in the low range of CRP concentrations in peripheral blood [17]. Several hsCRP assays have been developed with improved sensitivity and precision at low concentrations of CRP with the aim to detect subclinical inflammation [17]. Indeed, a high sensitivity of the assay may be of great importance to detect low grade inflammation in plasma. Therefore, we tested the hypothesis that in plasma a high sensitivity CRP assay (hsCRP assay) is more accurate than a classical CRP assay (classical CRP) to detect low grade inflammation in plasma of women with endometriosis.

The development of a reliable and accurate non-invasive diagnosis of endometriosis is one of the hot topics in endometriosis research. A clinically useful, non-invasive diagnostic test would have a groundbreaking impact on the patients' quality of life, on the efficacy of the available treatments as well as on the financial aspects of the disease [18].

The diagnostic accuracy of a test is commonly measured by using a ROC (receiver operating characteristic) curve analysis. When two different tests are compared, results are usually described as one test to be more sensitive or specific than the other. However, such results have only a descriptive character [19] and it is necessary to analyze statistically the performance (sensitivity and specificity) of both tests, and this analysis can impact medical decision making [19]. In this paper we compared the diagnostic performance of the hsCRP assay and the classical CRP assay to detect low grade inflammation in plasma of women with endometriosis.

## Methods

This study was approved by the Commission for Medical Ethics of the Leuven University Hospitals. Plasma samples were collected for the Endometriosis research Biobank after obtaining written informed consent from women undergoing laparoscopic surgery for subfertility with or without pain at the Leuven University Fertility Centre (LUFC) since 1999. Plasma samples were collected prior to anaesthesia induction in EDTA tubes, centrifuged at 3000 rpm for 10 minutes at 4°C, aliquoted, labelled and stored at -80°C till analysis. The time interval between sample collection and storage in the -80°C freezer was maximum 1 hour. For each patient, relevant information (e.g. date of collection, identification code, clinicopathological data) was entered in the electronic biobank database of the LUFC.

The following exclusion criteria were used: samples collected from women who were on hormonal medication at the time of collection, who had been operated within 6 months prior to the time of collection, or who had other pelvic inflammatory disease or general diseases at the time of collection.

A total of 295 plasma samples were selected from 204 women with laparoscopic confirmed endometriosis (ASRM stage I-II, n = 135; ASRM stage III-IV, n = 69) and 91 women with laparoscopic excluded endometriosis at the time of laparoscopic surgery for subfertility with or without pain at the LUFC during the menstrual (n = 60), luteal (n = 116) and follicular phase (n = 119) of the cycle (Table 1).

**Table 1:** Distribution of study samples according to stage of endometriosis and menstrual cycle phase

Cycle phase	Controls	Stage I-II	Stage III-IV
Menstrual	19	26	15
Follicular	36	60	23
Luteal	36	49	31
Total per Stage	91	135	69
TOTAL IN STUDY	295		

A power calculation was not done as this was an exploratory study using plasma samples obtained from our endometriosis research biobank. Most samples included in this study have been used in the previously published study of our group [14] where we evaluated 6 different potential plasma biomarkers (including hsCRP but not classical CRP) with the aim to develop a diagnostic panel for a non-invasive test for endometriosis.

In each sample, the level of CRP was measured twice using 2 different methods: the classical CRP assay (referred to as CRP), using an automated CRPLX Tina-quant C-Reactive Protein (Latex) assay (Roche, Vilvoorde, Belgium) and the high sensitivity CRP assay (referred to as hsCRP), using a (Latex) HS Tina-quant C-Reactive protein (latex) high sensitive assay (Roche, Vilvoorde, Belgium). Both assays are based on the principle of particle-enhanced immunological agglutination and were performed on a Roche Modular P instrument (Roche, Vilvoorde, Belgium) at the central laboratories of the University Hospitals Leuven (Gasthuisberg, Leuven). Briefly, anti-CRP antibodies coupled to latex microparticles react with CRP in the sample to form an antigen/antibody complex leading to agglutination causing turbidity of the reaction mixture, which is proportional to the CRP concentration and measured quantitatively.

The lower detection limit of the classical CRP assay and the hsCRP assay was 0.425 mg/L and 0.03 mg/L respectively. The functional sensitivity of the classical CRP assay and the hsCRP assay was 0.88 mg/L and 0.11 mg/L respectively.

For hsCRP, the within-run precision was 1.34% CV at 0.55 mg/dl and 0.28% CV at 12.36 mg/dl. Total imprecision was 5.70% CV and 2.51% CV at those concentration levels.

For CRP, the within-run precision was 2.5% CV at 5.76 mg/L and 0.76% CV at 150.1 mg/L. Total imprecision was 2.53% CV and 1.8% CV at those concentration levels.

Data are presented as median and range. A p value < 0.05 was considered statistically significant. Samples with plasma CRP levels below the detection limit of the assay were included in statistical analysis using the value corresponding with lower detection limit (LLD). Differences in CRP levels in women with and without endometriosis were evaluated using the Mann-Whitney test (endometriosis versus controls) and the Kruskal-Wallis test with post hoc Dunn analysis (minimal-mild endometriosis versus moderate-severe endometriosis versus controls).

The ROC curve analysis was performed to determine the diagnostic performance for both the classical CRP assay and the hsCRP assay separately. The area-under-the-curve (AUC) is a relative measure of the diagnostic accuracy and allows comparison of the diagnostic accuracy of different tests [20]. In our study the AUC was calculated and evaluated based on previously published guidelines and definitions [20,21]. The clinical value of a laboratory test with AUC values between 0 and 0.5, 0.5-0.7, 0.7-0.9, or > 0.9 can be defined as zero, limited, moderate, and high, respectively [20]. The ROC curves were compared by using Analyze-IT software for Microsoft Excel (Analyse-it Software, Ltd; Leeds, United Kingdom). The Mc Nemar test for correlated proportions was used to check differences in sensitivity between hsCRP and CRP tests as described before [19,22].

The numbers of plasma samples with detectable CRP levels between both assays were compared by using Chi-square test. Correlation analysis was performed by calculating the Spearman coefficient of correlation. The Fisher r to z transformation test was applied to assess the significance of the difference between two Spearman correlation coefficients. Bland-Altman analysis was performed to assess the agreement between two methods.

## Results

### CRP plasma levels: comparison between classical CRP assay and hsCRP assay in women with endometriosis and controls (Table 2)

#### Women with endometriosis versus controls

**Table 2:** Plasma hsCRP and CRP levels according to the cycle phase and disease stage

	All Phases	All phases	Luteal phase	Luteal phase	Follicular phase	Follicular phase	Menstrual phase	Menstrual phase
	CRP (mg/L)	hsCRP(mg/L)	CRP(mg/L)	hsCRP(mg/L)	CRP(mg/L)	hsCRP(mg/L)	CRP(mg/L)	hsCRP(mg/L)
Controls	0.425	0.62	0.425	0.54	0.425	0.62	0.425	0.75
	(0.425-14.6)	(0.11-15.03)	(0.425-6.3)	(0.11-7.15)	(0.425-10.3)	(0.18-10.59)	(0.425-14.6)	(0.27-15.03)
Stage I-IV	0.425	0.88	0.425	0.89	0.425	0.64	0.425	0.95
	(0.425-31.6)	(0.06-37.22)	(0.425-24.9)	(0.12-27.23)	(0.425-13.0)	(0.06-14.22)	(0.425-31.6)	(0.6-37.22)
P values (Mann-Whitney)	0.12	0.06	0.1	0.008	0.9	0.89	0.33	0.67
Stage I-II	0.425	0.64	0.425	0.66	0.425	0.49	0.425	0.87
	(0.425-31.6)	(0.06-37.22)	(0.425-13.1)	(0.12-14.14)	(0.425-5.6)	(0.06-6.6)	(0.425-31.6)	(0.15-37.22)
Stage III-IV	1.3	1.35	1.6	1.42	1	1.26	1.5	1.56
	(0.425-30.5)	(0.23-34.78)	(0.425-24.9)	(0.23-27.23)	(0.425-13.0)	(0.32-14.22)	(0.425-30.5)	(0.53-34.78)
P values (Kruskal-Wallis)	< 0.0001	< 0.0001	0.0033	0.0005	0.038	0.0025	0.29	0.19

#### Overall analysis (all cycle phases combined)

When compared to controls, CRP plasma levels were comparable (p = 0.12) or not-significantly elevated (p = 0.06) in women with endometriosis using either the classical CRP assay or the hsCRP assay, respectively (Table 2).

#### Separate analysis according to cycle phase (menstrual, follicular, luteal)

Significantly increased CRP plasma levels were found in women with endometriosis when compared to controls when the hsCRP assay was used in samples obtained during the luteal phase (p = 0.008), but not in samples obtained during follicular or menstrual phase (Table 2). No differences in CRP levels according to cycle phase were observed when the classical CRP assay was used (Table 2).

### Women with minimal-mild endometriosis or moderate-severe endometriosis versus controls

#### Overall analysis (all cycle phases combined)

The CRP plasma levels were higher in women with stage III-IV of endometriosis than in women with stage I-II endometriosis or than in controls using either the classical CRP assay (p < 0.0001 or p < 0.0001, respectively) or the hsCRP assay (p < 0.0001 or p < 0.0001, respectively).

#### Separate analysis according to cycle phase (menstrual, follicular, luteal)

When compared to controls, increased CRP plasma levels were found in women with stage III-IV of endometriosis using either the classical CRP assay or the hsCRP assay during both the luteal phase (p = 0.0033 or p = 0.0005, respectively) and the follicular phase (p = 0.038 or p = 0.0025, respectively) but not during the menstrual phase (Table 2).

### Diagnostic performance of hsCRP versus CRP

**Figure 1:**
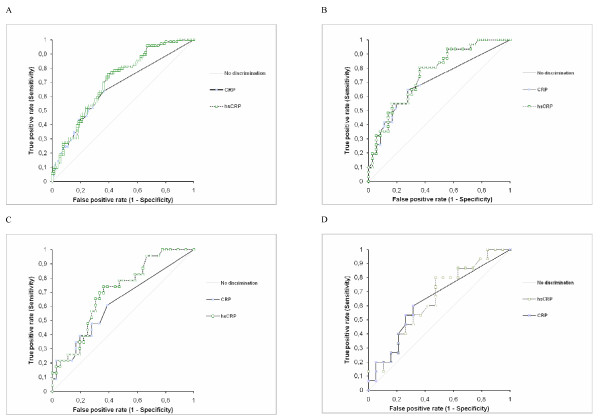
**ROC of hsCRP and CRP for prediction of moderate-severe endometriosis**. A: In an overall analysis (all cycle phases combined). The AUC was significantly higher for hsCRP analysis than for classical CRP analysis for the diagnosis of moderate-severe endometriosis in an overall analysis (all cycle phases combined, p = 0.018). B: In a luteal cycle phase. The AUC values of hsCRP and CRP were not significantly different (p = 0.065). C: In a follicular cycle phase. The AUC was higher for hsCRP analysis than for classical CRP analysis with borderline significance (p = 0.047). D: In a menstrual cycle phase. The AUC values of hsCRP and CRP were not significantly different (p = 0.83).

ROC curve analysis was performed for the classical CRP assay and the hsCRP assay separately to identify the discriminative power of the tests. Diagnostic performance was calculated at the optimal cut-off value (based on the highest sum of sensitivity and specificity). Table 3 illustrates the data for all stages of endometriosis combined and for minimal-mild and moderate-severe stages separately (overall data for all cycle phases combined and separate data according to phase of the menstrual cycle). Figure 1A-D illustrates the data for moderate-severe endometriosis (overall data for all cycle phases combined and separate data according to phase of the menstrual cycle). The area under the ROC curves (AUC) were compared as described previously [23]. Superior diagnostic performance (higher AUC) was demonstrated for the hsCRP assay when compared to the classical CRP assay (Table 3; Figure 1A-D). Indeed, the AUC was significantly higher for hsCRP analysis than for classical CRP analysis for the diagnosis of moderate-severe endometriosis in an overall analysis (all cycle phases combined, p = 0.018) and for the diagnosis of minimal-severe endometriosis during the luteal phase of the cycle only (p = 0.037). The highest discriminative ability for the diagnosis of endometriosis was obtained using the hsCRP assay during the luteal phase, especially for moderate -severe endometriosis (AUC 0.76, Table 3; Figure 1B). At a cut-off plasma level of hsCRP > 0.71 mg/L, moderate-severe stages of endometriosis were diagnosed with sensitivity of 80.65% and specificity of 63.89% during the luteal phase of the menstrual cycle. Using a similar cut-off value for CRP analyzed by the classical method during the same luteal phase, moderate-severe endometriosis was diagnosed (AUC 0.7, Table 3; Figure 1B) with lower sensitivity (67.7%) and comparable specificity (63.9%). However these differences in sensitivity were not statistically significant (p = 0.125 (two-tail) or 0.0625 (one-tail)).

**Table 3:** ROC curve analysis for CRP and hsCRP

	AUC	Cut-off	Sensitivity%/Specificity%	AUC	Cut-off	Sensitivity %/Specificity%
	CRP	CRP	CRP	hsCRP	hsCRP	hsCRP
		(mg/L)			(mg/L)	
All Phases						
Controls vs Stage I-IV	0.55	> 0.71	45.6/63.7	0.57	> 0.62	61.8/50.6
Controls vs Stage I-II	0.50	N/A	N/A	0.51	N/A	N/A
Controls vs Stage III-IV	0.66	> 0.71	63.8/63.7	0.71	> 0.72	78.3/56.0
Luteal phase						
Controls vs Stage I-IV	0.59	> 0.71	48.8/63.9	0.65	> 0.70	58.9/63.9
Controls vs Stage I-II	0.52	N/A	N/A	0.59	> 0.54	57/50
Controls vs Stage III-IV	0.70	> 0.71	67.7/63.9	0.76	> 0.71	80.7/63.9
Follicular phase						
Controls vs Stage I-IV	0.50	> 0.71	41.0/61.1	0.51	> 0.61	54.2/50
Controls vs Stage I-II	0,54	N/A	N/A	0.57	N/A	N/A
Controls vs Stage III-IV	0.62	> 0.71	60.9/61.1	0.70	> 0.66	78.3/52.8
Menstrual phase						
Controls vs Stage I-IV	0.57	> 0.71	49.0/68.0	0.54	> 0.73	68.3/47.4
Controls vs Stage I-II	0.53	N/A	N/A	0.53	N/A	N/A
Controls vs Stage III-IV	0.63	> 0.71	60.0/68.4	0.65	> 0.78	80.0/52.6

N/A- not applicable.

Due to comparable plasma CRP and hsCRP levels between women with stage I-II endometriosis and controls (Table 2), the ROC curve analysis was not useful and it was not possible to develop the optimal cut-off point for the diagnosis of stage I-II endometriosis (Table 3).

### Determination of CRP in plasma: Comparison of classical CRP and hsCRP assays

The number of plasma samples with detectable CRP was significantly higher (295/295 or 100%) using the hsCRP assay when compared to the classical CRP assay (126/295 or 42.7%) (p < 0.0001). Comparison of the CRP plasma levels between the classical CRP assay and the hsCRP assay showed a good Spearman correlation coefficient, ranging between 0.74 and 0.997 (Table 4), which was significantly higher (p < 0.001) in the endometriosis group (0.996) than in the control group (0.74). The bias and corresponding limits of agreement between two CRP tests are listed in Table 4.

**Table 4:** Comparison of two methods for the measurement of CRP in plasma

	Total	Controls	Stage I-IV	Stage I-II	Stage III-IV
CRP mg/L	0.425	0.425	0.425	0.425	1.3
	(0.425-31.6)	(0.425-14.6)	(0.425-31.6)	(0.425-31.6)	(0.425-30.5)
hsCRP mg/L	0.76 (0.06-37.22)	0.62 (0.11-15.03)	0.88 (0.06-37.22)	0.64 (0.06-37.22)	1.35 (0.23-34.78)
P values (Mann-Whitney)	0.92	0.77	0.759	0.68	0.46
Correlation coefficient (Spearman)	0.857	0.74	0.996	0.997	0.996
Bias	-0.15	-0.06	-0.19	-0.096	-0.38
SD of Bias	0.87	1.18	0.69	0.54	0.88
95% Limits of agreement	-1.85 and 1.55	-2.37 and 2.56	-0.56 and 1.15	-1.16 and 0.97	-2.10 and 1.35

## Discussion

To the best of our knowledge this is the first study which evaluated and compared the diagnostic performance of hsCRP and classical CRP assays in endometriosis based on the comparison of ROC curves and diagnostic sensitivities of these tests, as recommended by previous investigators [19,24,25].

In this study we demonstrated that CRP plasma concentrations within the normal reference range (between undetectable and 10 mg/L), as detected by the hsCRP assay, confirm the presence of subclinical inflammation in patients with endometriosis.

The concentrations of CRP levels in healthy adults are normally less than 10 mg/L and serum levels of CRP may increase over 1000-fold following inflammation or infection [6,26,27]. In clinical practise only the levels of CRP above the reference interval are considered as being of clinical significant [6]. However, CRP concentrations within the normal adult reference range provide valuable information of chronic inflammatory processes like Helicobacter pylori and Chlamydia pneumonia [6,28] and also endometriosis, as demonstrated in our study. Routine CRP assays often do not quantify CRP concentrations below the upper normal limit that may be relevant to detect subclinical inflammation which may occur early during disease development [29]. Such quantification is possible using high sensitivity CRP assays, based on the principle of particle-enhanced immunological agglutination or enzyme-linked immunosorbent assay [30].

In our study the hsCRP assay was superior to the classical CRP assay for the detection of subclinical inflammation in plasma of endometriosis patients. Our results are in line with the observations of other investigators [29,31], who provided evidence for an additional value of hsCRP in the clinical assessment of patients with Wegener's Granulomatosis, ankylosing spondylitis and non-radiographic axial spondyloarthritis [29,31]. These authors reported that hsCRP but not CRP levels, were higher in sera from patients who subsequently relapsed versus those who did not, indicating patients at risk [31]. In the mentioned studies, hsCRP levels were more closely associated with disease activity than CRP levels [29,31].

In our study, the hsCRP assay was superior to the classical CRP assay with respect to the proportion of plasma samples with detectable levels of CRP (100% versus 43%) and to its diagnostic performance (AUC, ROC curves, sensitivity). The diagnostic performance of hsCRP was superior to the diagnostic performance of classical CRP to distinguish endometriosis from controls (especially stage III-IV during the luteal cycle phase) (Table 3). Indeed the AUC was significantly higher after hsCRP analysis than after classical CRP analysis for the diagnosis of moderate-severe endometriosis in combined analysis (all cycle phases combined) and for minimal-severe endometriosis (during the luteal phase of the cycle only). The optimum cut-off value of plasma CRP > 0.71 mg/L obtained during the luteal phase of the menstrual cycle could predict nearly 13% more patients with moderate-severe endometriosis using the hsCRP assay when compared with the classical CRP assay. This is an interesting observation since our cut-off value (0.71 mg/L) was similar to the lowest cut-off point used in the risk assessment algorithm in the primary prevention of Cardiovascular disease [6,32]. In that survey, a mild relative risk of future cardiovascular disease was determined using low plasma levels of hsCRP between 0.7 -1.1 mg/L [32]. Although both endometriosis and atherosclerosis are associated with oxidative stress [33], at present there is no evidence that women with endometriosis have an increased risk for cardiovascular disease and atherosclerosis.

Comparable plasma CRP levels between women with endometriosis and controls (Table 2) resulted in a low AUC (0.55, Table 3) demonstrating limited clinical value.

Plasma CRP and hsCRP levels were comparable in women with stage I-II of endometriosis and controls (Table 2), resulting in low AUCs (0.50-0.57, Table 3), that did not allow determination of any cut-off points, demonstrating no clinical value.

The strength of this study lies in the fact that comparison of the diagnostic performance of hsCRP and CRP assays was evaluated on a large number of patients with laparoscopically confirmed or excluded endometriosis during all phases of the menstrual cycle.

The rationale for separating the different phases of the menstrual cycle is justified by the following observations.

Firstly, according to QUADAS (Quality Assessment of Diagnostic Accuracy Studies) guidelines [15,16], samples should be collected at a consistent phase of the cycle and results should be corrected for the cycle phases. Secondly, according to a recently published systematic review, lack of correction for the phase of the menstrual cycle can explain the considerable variability between studies with respect to blood levels of biomarkers in women with endometriosis and controls [15]. For instance, such correction was absent in three of the nine reviewed papers investigating IL-6 as a biomarker for endometriosis, despite evidence that levels are known to change throughout the cycle [15,34].

Thirdly, CRP levels in peripheral blood have been reported to be significantly higher during the midcycle and luteal phase when compared to the follicular phase [35].

Comparison of our results with those from other studies reporting CRP levels in women with endometriosis is difficult (Table 5) due to differences in patient phenotype, patient number, type of peripheral blood (serum or plasma) and CRP methodology (variety of assays manufactured by a variety of companies) [11-14,36]. The sensitivity of the CRP assay is crucial for measurements of analytes with low concentrations. However, using a hsCRP assay, no differences were found in serum CRP levels between women with and without endometriosis in 2 studies [12,13], including a much smaller number of patients (n = 38 and n = 82, respectively) than in our study. The high correlation between hsCRP and classical CRP assays observed in our study (Table 4) confirms data reported before [13].

**Table 5:** Performance of CRP as a biomarker for endometriosis

Authors	Type of study	Controls	Endometriosis	Methodology	Results
Abrao et al., 1997	Case-control study	N = 15 Time 1 Menstrual phase μg/ml: 1.58 ± 0.31 Time 2: Proliferative phase μg/ml: 1.53 ± 0.33	N = 35 Stage I-II = 20 Stage III-IV = 15 Menstrual phase μg/ml: Stage I-II: 5.06 ± 1.32 Stage III-IV: 13.15 ± 2.55 Proliferative phase μg/ml: Stage I-II: 2.89 ± 0.59 Stage III-IV: 3.52 ± 0.41	Serum Laparoscopically confirmed or excluded endometriosis Menstrual and Proliferative phases of cycle Home-made ELISA	Difference: Time1-Time 2 Controls: 0.05 ± 0.38 Stage I-II: 2.17 ± 1.16 Stage III-IV: 9.63 ± 2.34 Increased levels of CRP in endometriosis (especially for stages III-IV)
Lermann et al., 2010	Prospective nonrandomized controlled trial	N = 34 CRP: 2.88 ± 2.79 ng/ml hsCRP: 2.48 ± 3.77 ng/ml	N = 48 Stage I: 7 Stage II: 5 Stage III: 18 Stage IV: 18 CRP: 3.54 ± 3.24 hsCRP: 3.61 ± 4.82	Serum Laparoscopically confirmed or excluded endometriosis Early Proliferative cycle phase CRP and hsCRP methods (details Not Available)	NS differences
Matarese et al., 2000	Case-control study	N = 15	N = 13 Stage I-II: 7 Stage III-IV: 6	Serum Laparoscopically confirmed or excluded endometriosis Proliferative and secretive cycle phase N Latex CRP monokit (Bering Nephelometer Systems)	CRP serum concentrations were less than 3.5 mg/L in all subjects
Mihalyi et al., 2010	Case-control study	N = 93 All phases: 0.64 (0.11-15.03) mg/L Secretory phase 0.56 (0.11-14.14) mg/L	N = 201 All phases Stage III-IV: 1.35 (0.23-34.78) mg/L Secretory phase Stage I-IV: 0.88 (0.12-27.23) mg/L	EDTA Plasma Laparoscopically confirmed or excluded endometriosis Menstrual, Proliferative and Secretory phases hsCRP method: HS Tina-quant CRP (latex) hs assay (Roche, Vilvoorde, Belgium)	Significant increase in Stage III-IV (independent of cycle phase and in the luteal phase (p < 0.0001; p = 0.001respectively) Significant increase in Stage I-IV of endometriosis in the luteal phase (p = 0.03)
Xavier et al., 2005	Case-control study	N = 13 Early proliferative: 0.68 (0.19-2.22) mg/L Late proliferative: 0.59 (0.21-1.28) mg/L Early secretory: 0.90 (0.42-2.31) mg/L Late secretory: 0.87 (0.22-1.52) mg/L	N = 25 Stage III-IV Early proliferative: 2.29 (0.70-3.54) mg/L Late proliferative: 1.10 (0.30-2.06) mg/L Early secretory: 1.09 (0.39-2.62) mg/L Late secretory: 0.60 (0.28-3.19) mg/L	Serum Early and late Proliferative phase Early and late Secretory phase hsCRP two-site homemade ELISA	NS differences

In conclusion, the hsCRP assay was superior to the classical CRP assay for the detection of low CRP levels indicating subclinical inflammation in plasma of endometriosis patients. Diagnostic performance of hsCRP was superior to classical CRP in women with moderate-severe endometriosis. However, CRP is not useful for diagnosis of early stages of endometriosis.

## List of abbreviations

CRP: C-reactive protein; hs: high sensitivity; ROC: receiver operating characteristic; LUFC: Leuven University Fertility Centre; LLD: lower limit of detection; AUC: areas under the ROC curves.

## Competing interests

The authors declare that they have no competing interests.

## Authors' contributions

Study concept and design: AV, CMK, XB, TD; Acquisition of data: AV, CMK, XB, CM, KP, CT, TD; Analysis and interpretation of data: AV, XB, CMK, AF, TD; Drafting of the manuscript: AV, XB, AF, TD; Critical revision of the manuscript for important intellectual content: AV, XB, AF, CMK, CM, KP, CT, TD. Prof. TM D'Hooghe had full access to all the data in the study and had final responsibility for the decision to submit for publication. All authors read and approved the final manuscript.

## References

[B1] Mihalyi A Kyama CM Simsa P Debrock S Mwenda JM D'Hooghe TM Role of immunologic and inflammatory factors in the development of endometriosis: indications for treatment strategies Therapy 2005 2 623 639 10.2217/14750708.2.4.623

[B2] D'Hooghe TM Debrock S Hill JA Meuleman C Endometriosis and subfertility: Is the relationship resolved? Semin Reprod Med 2003 21 243 254 12917793 10.1055/s-2003-41330

[B3] Kyama CM Debrock S Mwenda JM D'Hooghe TM Potential involvement of the immune system in the development of endometriosis Reprod Biol Endocrinol 2003 123 1 9 10.1186/1477-7827-1-123 PMC305339 14651748

[B4] Matalliotakis IM Goumenou AG Koumantakis GE Neonaki MA Koumantakis EE Dionyssopoulou E Athanassakis I Vassiliadis S Serum concentrations of growth factors in women with and without endometriosis: the action of anti-endometriosis medicines Int Immunopharmacol 2003 3 81 89 10.1016/S1567-5769(02)00216-3 12538037

[B5] Vigano P Endometriosis: epidemiology and aetiological factors Best Pract Res Clinical Obstet Gynaecl 2004 18 177 200 10.1016/j.bpobgyn.2004.01.007 15157637

[B6] Ledue TB Rifai N High sensitivity immunoassays for C-reactive protein: Promises and pitfalls Clin Chem Lab Med 2001 39 1171 1176 10.1515/CCLM.2001.185 11831635

[B7] Agic A Xu H Finas D Banz C Diedrich K Hornung D Is endometriosis associated with Systemic Subclinical Inflammation? Gynecol Obstet Invest 2006 62 139 147 10.1159/000093121 16679772

[B8] Rifai N Tracy RP Ridker PM Clinical efficacy of an automated high-sensitivity C-reactive protein assay Clin Chem 1999 45 2136 2141 10585345

[B9] Verma S Yeh ET C-reactive protein and atherothrombosis--beyond a biomarker: an actual partaker of lesion formation Am J Physiol Regul Integr Comp Physiol 2003 285 R1250 1252 14557241 10.1152/ajpregu.00170.2003

[B10] Black S Kushner I Samols D C-reactive protein J Biol Chem 2004 279 48487 48490 10.1074/jbc.R400025200 15337754

[B11] Abrao MS Podgaec S Filho BM Ramos LO Pinotti JA de Oliveira RM The use of biochemical markers in the diagnosis of pelvic endometriosis Hum Reprod 1997 12 2523 2527 10.1093/humrep/12.11.2523 9436699

[B12] Xavier P Belo L Beires J Rebelo I Lunet N Barros H Serum levels of VEGF and TNF-a And their association with C-reactive protein in patients with endometriosis Arch Gynecol Obstet 2006 273 227 231 10.1007/s00404-005-0080-4 16208475

[B13] Lermann J Mueller A Korber F Oppelt P Beckmann R Dittrich R Renner SP Evaluation of high-sensitivity reactive protein in comparison with C-reactive protein as biochemical serum marker in women with endometriosis Fertil Steril 2010 93 2125 2129 10.1016/j.fertnstert.2009.01.072 19232412

[B14] Mihalyi A Gevaert O Kyama CM Simsa P Pochet N De Smet F De Moor B Meuleman C Billen J Blanckaert N Non-invasive diagnosis of endometriosis based on a combined analysis of six plasma biomarkers Hum Reprod 2010 25 654 664 10.1093/humrep/dep425 20007161

[B15] May KE Conduit-Hulbert SA Villar J Kirtley S Kennedy SH Becker CM Peripheral biomarkers of endometriosis: a systematic review Hum Reprod Update 2010 16 651 674 10.1093/humupd/dmq009 PMC2953938 20462942

[B16] Whiting P Rutjes AW Reitsma JB Bossuyt PM Kleijnen J The development of QUADAS: a tool for the quality assessment of studies of diagnostic accuracy included in systematic reviews BMC Med Res Methodol 2003 3 25 10.1186/1471-2288-3-25 14606960 PMC305345

[B17] Roberts WL Moulton L Law TC Farrow G Cooper-Anderson M Savory J Rifai N Evaluation of nine automated high-sensitivity C-reactive protein methods: implications for clinical and epidemiological applications Part 2 Clin Chem 2001 47 418 425 11238291

[B18] D'Hooghe TM Mihalyi AM Simsa P Kyama CK Peeraer K De Loecker P Meeuwis L Segal L Meuleman C Why we need a non-invasive diagnostic test for minimal to mild endometriosis with a high sensitivity Editorial and Opinion Paper Gynecol Obstet Invest 2006 62 136 138 10.1159/000093120 16679771

[B19] Hawass NED Comparing the sensitivities and specificities of two diagnostic procedures performed on the same group of patients Br J Radiol 1997 70 360 366 9166071 10.1259/bjr.70.832.9166071

[B20] Bossuyt X Clinical performance characteristics of a laboratory test. A practical approach in the autoimmune laboratory Autoimmunity Reviews 2009 8 543 548 10.1016/j.autrev.2009.01.013 19200856

[B21] Akobeng A Understanding diagnostic tests 3: Receiver operating characteristic curves Acta Paediatr 2007 96 644 647 10.1111/j.1651-2227.2006.00178.x 17376185

[B22] Waaseth M Bakken K Dumeaux V Olsen K Rylander C Figenschau Y Lund E Hormone replacement therapy use and plasma levels of sex hormones in the Norwegian Women and Cancer Postgenome Cohort -a cross-sectional analysis BMC Womens Health 2008 8 1 11 10.1186/1472-6874-8-1 18194511 PMC2254595

[B23] De Long ER De Long DM Clarke-Pearson DL Comparing the areas under two or more correlated receiver operating characteristic curves: a nonparametric approach Biometrics 1988 44 837 845 10.2307/2531595 3203132

[B24] Royal HD McNeil BJ Quantitative analysis in clinical nuclear medicine Clinical Nuclear Medicine 1983 1 London: Chapman & Hall Ltd 473 479

[B25] Glantz AS Primer of Bio-statistics 1992 3 New York: Mc Graw Hill, Inc 32 33

[B26] Gewurz H Biology of C-reactive protein and the acute phase response Hosp Pract 1982 17 67 71 10.1080/21548331.1982.11702332 7201970

[B27] Singer JM Plotz CM Bader E Elster SK The latex-fixation test. III. Agglutination test for C-reactive protein and comparison with the capillary precipitin method Am J Clin Pathol 1957 28 611 617 13508578 10.1093/ajcp/28.6.611

[B28] Patel P Mendall M Carrington DJ Strachan D Leatham E Molineaux N Levy J Blakeston C Seymour CA Camm AJ Association of Helicobacter pylori and Chlamidia pheumoniae infection with coronary heart disease and cardiovascular risk factor Br Med J 1995 311 711 714 10.1136/bmj.311.7007.711 PMC2550716 7549683

[B29] Poddubnyy DA Rudwaleit M Listing J Braun J Sieper J Comparison of a high sensitivity and standard C reactive protein measurement in patients with ankylosing spondylitis and non-radiographic axial spondyloarthritis Annals of the Rheumatic Diseases 2010 69 1338 1341 10.1136/ard.2009.120139 20498207

[B30] Malek A Bersinger NA Di Santo S Mueller MD Sager R Schneider H Ghezzi F Karousou E Passi A De Luca G Raio L C-Reactive Protein Production in Term Human Placental Tissue Placenta 2006 27 619 625 10.1016/j.placenta.2005.05.009 16026834

[B31] Kalsch AI Csernok E Munch D Birck R Yard BA Gross W Kalsch T Schmitt WH Use of Highly Sensitive C-Reactive Protein for Followup of Wegener's Granulomatosis Journal of Rheumatology 2010 37 2319 2325 10.3899/jrheum.100302 20716656

[B32] Ridker PM High-sensitivity C-reactive protein - Potential adjunct for global risk assessment in the primary prevention of cardiovascular disease Circulation 2001 103 1813 1818 11282915 10.1161/01.cir.103.13.1813

[B33] Pretta S Remorgida V Abbamonte LH Anserini P Ragni N Del Sette M Gandolfo C Ferrero S Atherosclerosis in women with endometriosis Eur J Obstet Gynecol Reprod Biol 2007 132 226 231 10.1016/j.ejogrb.2006.04.015 16682112

[B34] Angstwurm M Gartner R Ziegler-Heitbrock H Cyclic plasma IL-6 levels during normal menstrual cycle Cytokine 1997 9 370 374 10.1006/cyto.1996.0178 9195137

[B35] Jilma B Dirnberger E Löscher I Rumplmayr A Hildebrandt J Eichler H Stylianos K Wagner O Menstrual cycle-associated changes in blood levels of interleukin-6, α1 acid glycoprotein, and C-reactive protein Journal of Laboratory and Clinical Medicine 1997 130 69 75 10.1016/S0022-2143(97)90060-3 9242368

[B36] Matarese G Alviggi C Sanna V Howard JK Lord GM Carravetta C Fontana S Lechler RI Bloom SR De Placido G Increased leptin levels in serum and peritoneal fluid of patients with pelvic endometriosis J Clin Endocrinol Metab 2000 85 2483 2487 10.1210/jc.85.7.2483 10902797

